# Dopamine Signaling in *C. elegans* Is Mediated in Part by HLH-17-Dependent Regulation of Extracellular Dopamine Levels

**DOI:** 10.1534/g3.114.010819

**Published:** 2014-04-07

**Authors:** Chaquettea M. Felton, Casonya M. Johnson

**Affiliations:** Department of Biology, School of Arts and Sciences, Georgia State University, Atlanta, Georgia 30303

**Keywords:** reserpine, bupropion, fluoxetine, dopamine receptor, acetylcholine signaling

## Abstract

In *Caenorhabditis elegans*, the dopamine transporter DAT-1 regulates synaptic dopamine (DA) signaling by controlling extracellular DA levels. In *dat-1(ok157)* animals, DA is not taken back up presynaptically but instead reaches extrasynpatic sites, where it activates the dopamine receptor DOP-3 on choligeneric motor neurons and causes animals to become paralyzed in water. This phenotype is called swimming-induced paralysis (SWIP) and is dependent on *dat-1* and *dop-3*. Upstream regulators of *dat-1* and *dop-3* have yet to be described in *C. elegans*. In our previous studies, we defined a role for HLH-17 during dopamine response through its regulation of the dopamine receptors. Here we continue our characterization of the effects of HLH-17 on dopamine signaling. Our results suggest that HLH-17 acts downstream of dopamine synthesis to regulate the expression of *dop-3* and *dat-1*. First, we show that *hlh-17* animals display a SWIP phenotype that is consistent with its regulation of *dop-3* and *dat-1*. Second, we show that this behavior is enhanced by treatment with the dopamine reuptake inhibitor, bupropion, in both *hlh-17* and *dat-1* animals, a result suggesting that SWIP behavior is regulated via a mechanism that is both dependent on and independent of DAT-1. Third, and finally, we show that although the SWIP phenotype of *hlh-17* animals is unresponsive to the dopamine agonist, reserpine, and to the antidepressant, fluoxetine, *hlh-17* animals are not defective in acetylcholine signaling. Taken together, our work suggests that HLH-17 is required to maintain normal levels of dopamine in the synaptic cleft through its regulation of *dop-3* and *dat-1*.

In *Caenorhabditis elegans* and other multicellular organisms, basic helix-loop-helix (bHLH) proteins coordinate a number of developmental events, including myogenesis (Chen *et al.* 1994), organ morphogenesis (Tamai and Nishiwaki 2007), and mesodermal development (Harfe *et al.* 1998). These proteins also have vital functions during neurogenesis (Hallam *et al.* 2000; Krause *et al.* 1997). For example, the proneural protein HLH-14 is required to generate multiple neurons stemming from a variety cell lineage types, while HLH-3 is needed for the differentiation of hermaphrodite-specific motor neurons (Doonan *et al.* 2008; Frank *et al.* 2003; Poole *et al.* 2011). HLH-17 is the *C. elegans* homolog of the mammalian proneural family Olig (Ligon *et al.* 2006; Zhou and Anderson 2002) but does not appear to play a role in neuronal specification during embryogenesis (Yoshimura *et al.* 2008). Our previous studies instead demonstrated that HLH-17 is required for normal behavioral responses to dopamine signaling (Felton and Johnson, 2011).

In vertebrates and invertebrates, dopamine signaling is associated with motivation, recognition and reward, memory and adaptation, hormonal regulation, and motor control. In humans, imbalances in dopamine signaling are associated with many neurological diseases, including Parkinson disease, Alzheimer disease, ADHD, and substance abuse (Choi and Tarazi 2010; Middleton *et al.* 2007; Xie *et al.* 2010). Dopamine signaling in *C. elegans* involves many of the same molecules as in mammals (Chase and Koelle 2007). For example, dopamine is synthesized by the tyrosine hydroxylase enzyme CAT-2. On synthesis, dopamine is sequestered in presynaptic storage vesicles by the vesicular monoamine transporter CAT-1, where it remains until being released into the presynaptic cleft in response to a stimulus. Once in the synapse, dopamine binds to and activates D1-like (DOP-1) and D-2 like receptors (DOP-2 and DOP-3) that are positioned either pre-, post-, or extra-synaptically. Unbound dopamine is taken back up into the presynaptic cell via reuptake by the dopamine transporter DAT-1.

HLH-17 is expressed in the glia-like cells surrounding the CEP dopaminergic neurons (McMiller and Johnson 2005) and in the sheath or socket cells of the inner labia and outer labia (Yoshimura *et al.* 2008). Our previous data revealed that HLH-17 affects dopamine signaling through the DOP-1, DOP-2, and DOP-3 receptors as shown by the impaired response of *hlh-17(ns204)* animals to endogenous and exogenous dopamine. The *hlh-17(ns204)* animals also have reduced levels of the *dop-3* and *dop-1* mRNAs and phenocopy *dop-3* hypomorhs (Chase *et al.* 2004; Felton and Johnson 2011). Together, these data suggest that HLH-17 functions upstream of the dopamine receptor genes and that the loss of *hlh-17* causes a reduction in dopamine receptor activity.

Here we continue our characterization of the role of HLH-17 in dopamine signaling. Our data suggest that HLH-17 influences dopamine-dependent behaviors by regulating genes that mediate levels of extracellular dopamine. The *dat-1* mRNA levels are reduced, but not eliminated, in *hlh-17(ns204)* animals. Furthermore, *hlh-17(ns204)* animals display swimming-induced paralysis (SWIP) behavior in water that is an intermediate between the behavior in *dat-1* animals and in wild-type animals and that is enhanced by treatment with the dopamine reuptake inhibitor, bupropion. We show that a null allele of *dop-3* completely suppresses the SWIP phenotype of *hlh-17* animals, supporting previous data that HLH-17 acts upstream of DOP-3. Surprisingly, the SWIP phenotype of *hlh-17* animals is unaffected by treatment with the VMAT inhibitor reserpine or with the serotonin reuptake inhibitor, fluoxetine; however, this unresponsiveness is not due to reduced acetylcholine signaling. Taken together, our results suggest that HLH-17 influences extracellular dopamine levels in *C. elegans*, in part by its regulation of the dopamine receptors and the dopamine transporter.

## Materials and Methods

### Nematode strains and maintenance

The following strains were used in this study: wild-type: Bristol strain (N2); RM2702 [*dat-1(ok157)*]; OS2649: [*hlh-17(ns204*)]; and LX705 [*dop-1 (vs100) dop-3 (vs106)*]. OS2649 was a gift from Dr. S. Shaham. The strains CMJ2003 [*hlh-17(ns204)*; *dat-1(ok157)*] and CMJ2004 [*hlh-17(ns204)*; *dop-1(vs100) dop-3(vs106)*] were generated using traditional crossing techniques and the genotypes were confirmed by PCR. To generate CMJ2004, *hlh-17(ns204)* males were crossed with *dop-1(vs100) dop-3(vs106)* hermaphrodites, and the F1 males were backcrossed to *dop-1(vs100) dop-3(vs106)*. F2 hermaphrodites were separately cloned, and their progeny were genotyped by PCR. The strain CMJ2005 [*hlh-17(ns204)*; *dat-1(ok157)*; *dop-1(vs100) dop-3 (vs106)*] was generated by crossing CMJ2003 males with CMJ2004. F1 hermaphrodites were separately cloned, and their progeny were genotyped by PCR for *hlh-17* and *dat-1*. The progeny of hermaphrodites that were confirmed to be homozygous for both *hlh-17* and *dat-1* were then subcloned and their progeny were screened for homozygosity for *dop-3* by PCR and for rescue of SWIP behavior.

The transgene, cmjEx22, is a 6.2-kb genomic fragment consisting of 2 kbp upstream of the *hlh-17* translational start site, the entire *hlh-17* coding region, the SV40 nuclear localization signal (NLS), and 850 bp of the sequences coding for green fluorescent protein (GFP). The GFP sequences were amplified from pPD95.67 (a gift from A. Fire) using serial overlap PCR. Transgenic lines to rescue loss of *hlh-17* were produced by microinjecting the final PCR product (cmjEx22) into *hlh-17(ns204)* animals, along with the pCFJ90 [P*myo-2*::mCherry::unc-54utr] co-injection marker, using standard microinjection techniques(Rieckher *et al.* 2009), and is designated as CMJ2002 [*hlh-17(ns204)*; cmjEX22, pCFJ90(P*myo-2*::mCherry::unc54utr)].

Three separate lines (15.1, 15.3, and 3.1) were tested for rescue of SWIP, basal slowing response, and dopamine paralysis. All three lines were able to at least partially rescue each of the phenotypes tested; however, the degree of rescue for each line was specific to the phenotype tested.

Unless otherwise noted, strains were cultured on solid nematode growth media (NGM) containing OP50 at 20° using standard methods and synchronized cultures were prepared by hypochlorite treatment of gravid adults, as previously described (Brenner 1974). The following primers were used for genotyping: HLH17F: 5′-TCTGGGGACCCTCTCCTCG-3′; HLH17R: 5′-CGATTTTTGCTGCTAATGGGCAACAC-3′; DAT1F: 5′-CTATTCGGATATCTTGCCAATGCTATACC-3′; DAT1R: 5′-CTATTCGGATATCTTGCCAATGCTATACC-3′; DOP3F: 5′-CTATTCGGATATCTTGCCAATGCTATACC-3′; and DOP3R: 5′-CTAACTCACCAGAAAATCAGAAACTGC-3′.

### Gene expression analysis

Synchronized populations were collected at the L4 stage, pelleted, and frozen at −80°. Total RNA, cDNA synthesis, and real-time PCR were performed as previously described (Felton and Johnson 2011), except the cDNA was amplified from 1 µg of total RNA in 20 µL reactions. Real-time PCR was performed with Taqman Gene Expression Assays (Applied Biosystems/Invitrogen) using relative quantitation against glyceraldehyde 3-phosphate dehydrogenase (*gpd-3*) (Ce02616909_gH) as the endogenous control. The probe sets used were: *hlh-17*(Ce02616669_m1); *dat-1*(Ce02450896_g1); *cat-1*(Ce02495610_m1); *mod-5* (Ce02415245_m1); *dop-1* (Ce02494345_m1); *dop-2* (Ce02479824_m1) *dop-3*(Ce02496462_m1); *lev-8* (Ce02501240_g1); and unc-43 (Ce02458977_m1). Gene expression assays were performed in triplicate for at least three biological replicates.

### Behavioral assays

Assays for dopamine paralysis and basal slowing response were as previously described (Felton and Johnson 2011), except animals were assayed at the late L4 stage. For SWIP, approximately 10 L4-stage animals were placed in 150 µL of water in a single well of 48-well tissue culture plate (Cat #677180; CELLSTAR). After 20 min, animals were categorized as paralyzed if they failed to exert the normal thrashing behavior within a 20-sec time frame (McDonald *et al.* 2007). For SWIP assay conducted with inhibitors, animals were grown on NGM plates containing the appropriate drug [reserpine (0.6 mM; Cat #S1601), fluoxetine (145 µM; Cat #S1333), and bupropion (10 μM; Cat #S2452)] and then analyzed in water. All inhibitors were obtained from Selleckchem. Aldicarb-induced paralysis and levamisole-induced paralysis assays were conducted using standard protocols (Lewis *et al.* 1980; Nguyen *et al.* 1995; Mahoney *et al.* 2006) with some modifications. Plates containing aldicarb (1.0 mM; FisherSci #US-PST-940) or levamisole (0.2 mM; FisherSci #ICN15522805) were prepared 1 hr before use. Drugs were prepared as 100-mM stocks in 70% ethanol, diluted in sterile M9 buffer, added to NGM plates already seeded with OP50, to the appropriate concentration, and allowed to diffuse into the media for 1 hr. L4-stage animals were manually selected to confirm their age and moved to plates using a platinum wire and were examined every hour for a 5-hr to 6-hr period. Animals were categorized as paralyzed if they failed to move after prodding with a platinum wire.

## Results and Discussion

### HLH-17 functions upstream of the D2-like dopamine receptor DOP-3 to regulate behavioral responses to dopamine

**Figure 1:**
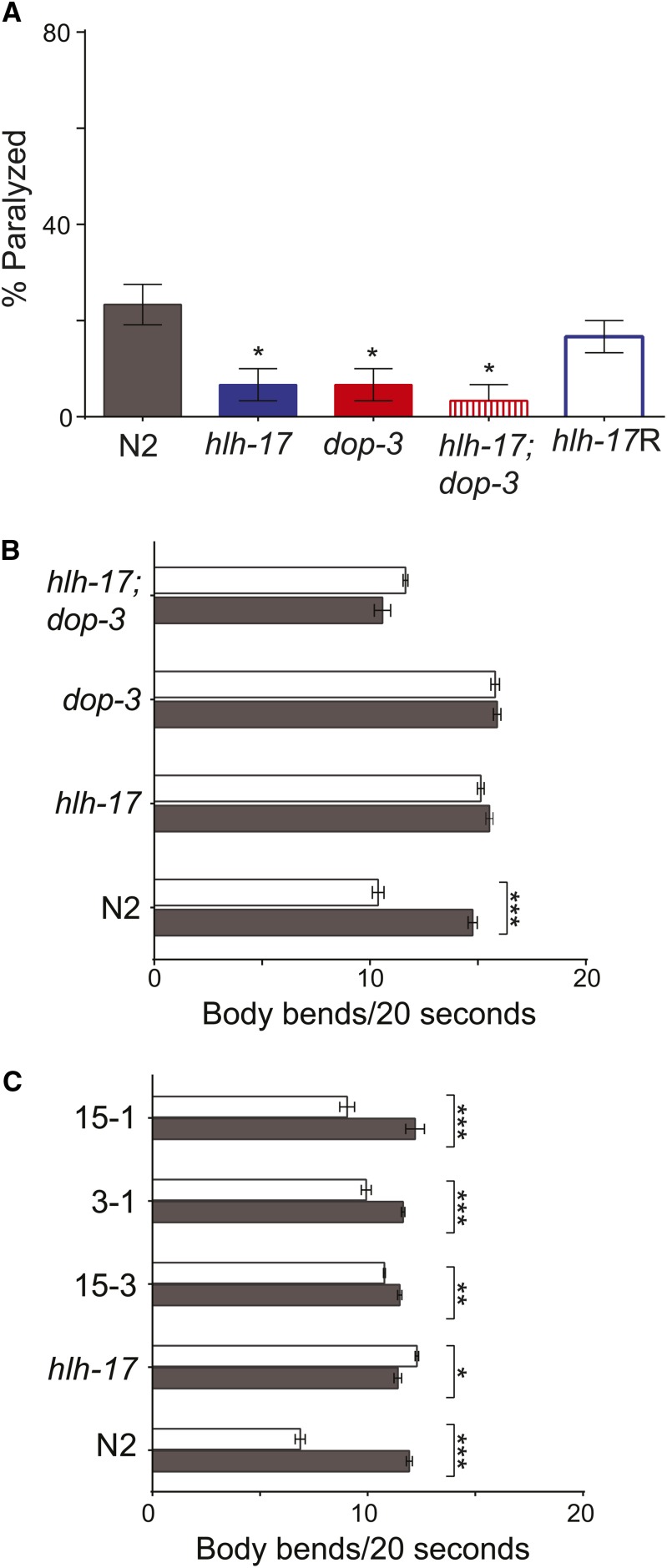
HLH-17 functions upstream of *dop-3* to regulate dopamine signaling. (A) DA-induced paralysis: *hlh-17(ns204)*, *dop-3 (vs106)*, and *hlh-17(ns204)*; *dop-3 (vs106)* animals are less sensitive than wild-type animals to 10 mM DA. Transgenic expression of HLH-17::GFP in *hlh-17(ns204)* animals rescues the DA-induced paralysis phenotype. The bar for *hlh-17*R represents the average measurements from three biological replicas of three independent lines. *Statistical significance when compared to wild-type, n = 10 animals/strain/rep for three biological replicas. (B) Basal slowing response: Well-fed wild-type animals, but not *hlh-17(ns204)*, *dop-3 (vs106)*, or *hlh-17(ns204)*; *dop-3 (vs106)* animals, move significantly slower in the presence of food (white bars) than in the absence of food (gray bars). (C) Transgenic expression of HLH-17::GFP rescues the basal slowing response of *hlh-17(ns204)* animals. Three independent lines, 15.3, 15-1, and 3.1, were assayed. In (B) and (C), five animals/rep/strain for a total of three biological replicas were assayed. Each animal was analyzed for three separate 20-sec intervals, so that the total number of observations was 15 observations/rep/strain. *P < 0.05; **P < 0.005; ***P < 0.0005.

The effects of dopamine signaling in *C. elegans* are mediated by the three heterotrimeric G-protein receptors, DOP-1, DOP-2, and DOP-3 (Missale *et al.* 1998). Our previous studies demonstrated that mRNA levels of these three receptors are reduced in *hlh-17(ns204)* animals and that *hlh-17(ns204)* animals phenocopy those carrying loss-of-function alleles of *dop-3* (Felton and Johnson 2011). As shown in Figure 1, and in our previous studies, fewer *hlh-17(ns204)* and *dop-3(vs106)* animals than wild-type animals were paralyzed after 40 min of exposure to 10 mM of exogenous DA, and both well-fed *hlh-17(ns204)* animals and well-fed *dop-3(vs106)* animals failed to exhibit the basal slowing response (BSR) when encountering a bacterial lawn. In this study, we used an extragenic, translational reporter for *hlh-17* to rescue the dopamine paralysis and basal slowing phenotypes of transgenic *hlh-17(ns204)* animals. This reporter was able to restore dopamine sensitivity and to enhance BSR, showing that the previously reported phenotypes are indeed a result of loss of HLH-17 (Figure 1). We previously reported that a transcriptional reporter for *hlh-17* drives expression in the glial-like, cephalic sheath cells of the dopaminergic neurons (McMiller and Johnson 2005), and others have detected weak *hlh-17* expression in the sheath or socket cells of the inner labia and outer labia (Yoshimura *et al.* 2008). The translational reporter used in this study was driven by the same promoter sequences and was similarly expressed (data not shown). This expression pattern weakly correlates with expression of the dopamine receptors in neuronal support cells of the head (Chase *et al.* 2004); therefore, we looked for genetic interactions between *hlh-17* and *dop-3*. As shown in Figure 1, the resistance to dopamine-induced paralysis and the BSR of *hlh-17(ns204)*; *dop-3(vs106)* are not significantly different from the resistance and slowing response phenotypes of *dop-3(vs106)* and *hlh-17(ns204)* animals (Figure 1) and are consistent with a model in which HLH-17 is functioning in the same genetic pathway as DOP-3 to modulate these behaviors. Taken together, we conclude that the influence of HLH-17 on behaviors that are mediated by dopamine occurs through the transcriptional regulation of *dop-3*. Our existing data suggest that this regulation is indirect; however, it is possible that the transcriptional and translational constructs used in our studies do not fully report the wild-type expression pattern for *hlh-17*. In fact, recent gene expression profiles from FACS sorted cells point to overlapping expression of *hlh-17* and *dop-3* in the dopamine neurons of late embryos, in panneuronal cells and the glutamate receptor neurons of L2-stage animals, and in the cephalic sheath of young adult animals (Spencer *et al.* 2011; see WormViz at http://www.vanderbilt.edu/wormdoc/wormmap/Worm*Viz*.html). Additionally, dopamine receptor genes are expressed in mammalian glial cells (Biedermann *et al.* 1995; Kuric *et al.* 2013) and further support the possibility that HLH-17 directly regulates as *dop-3* expression in the cephalic sheath and in selected neurons during *C. elegans* development.

### The *hlh-17* mutants are defective in clearing dopamine from the synaptic cleft

In our previous studies, the mRNA levels of genes required for dopamine synthesis, those encoding tyrosine hydroxylase gene (*cat-2*) and the aromatic amino acid decarboxylase (*bas-1*), were not affected by loss of *hlh-17*. This suggested that the presynaptic synthesis of dopamine is not compromised in *hlh-17(ns204)* animals. Additionally, exogenous dopamine failed to repress egg-laying in naive *hlh-17(ns204)* animals; however, exogenous dopamine was able to repress the stimulation of egg-laying by the neurotransmitter, serotonin (Felton and Johnson 2011). Although we did not further address the serotonin responsiveness of *hlh-17(ns204)* animals, this result suggested that some ability of *hlh-17(ns204)* animals to respond to exogenous dopamine may be mediated by the binding of the neurotransmitter to other non-dopaminergic receptors. For example, dopamine can bind with low affinity to a number of the neurotransmitter receptors involved in serotonin-stimulated egg-laying, including MOD-1, SER-1, SER-2, and SER-7 (Chase and Koelle 2007; Dempsey *et al.* 2005).

**Figure 2:**
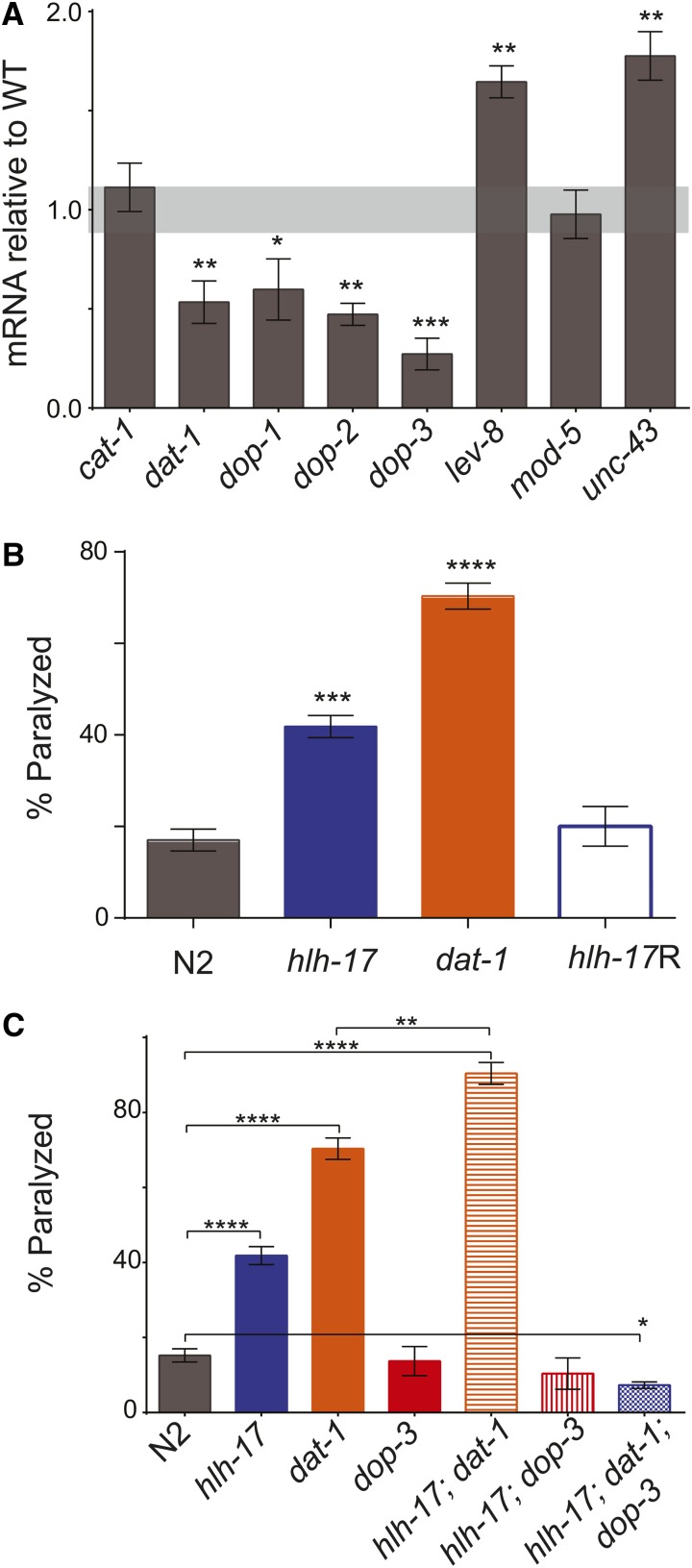
Loss-of *hlh-17* affects extracellular DA levels. (A) mRNA levels in L4-stage *hlh-17(ns204)* animals when normalized against mRNA levels in age-matched wild-type animals. Light gray shading represents wild-type range of expression (1.0 ± 0.115). The levels of *cat-1 and mod-5* mRNA are not significantly affected in *hlh-17(ns204)* animals. (B) *hlh-17(ns204)* animals demonstrate SWIP behavior that is an intermediate of the behavior in N2 and *dat-1(ok157)* animals, and that is rescued by transgenic expression of HLH-17::GFP. The bar for *hlh-17*R represents the average measurements from three biological replicas of three independent lines. For all strains except hlh-17R, n = 30 animals/rep/strain. For hlh-17R, n was equal to an average of at least 15 animals/line/biological rep (range, 12–26) because of differences in transmission frequency of the transgene. (C) SWIP phenotype in double mutant *hlh-17(ns204)*; *dat-1(ok157)* and *hlh-17(ns204)*; *dop-3(vs106)* animals is more similar to the phenotype in *dat-1(ok157)* and *dop-3(vs106)* animals, respectively, than in wild-type animals. The SWIP phenotype of *hlh-17(ns204)*; *dat-1(ok157)*; *dop-3(vs106)* animals is not significantly different from the SWIP phenotypes of *dop-3* or *hlh-17(ns204)*; *dop-3(vs106)* animals. n = 30 animals/rep/strain for three biological replicas. *P < 0.05; **P < 0.005; ***P < 0.0005; ****P < 0.0001.

To further define the role of HLH-17 during dopamine signaling, we measured the mRNA levels of the genes encoding the vesicular monoamine transporter (VMAT), *cat-1*, and the dopamine reuptake transporter, *dat-1*, in *hlh-17(ns204)* animals. As shown in Figure 2, *dat-1*, but not *cat-1*, mRNA levels, are decreased in *hlh-17(ns204)* animals. We also found that the mRNA levels for the dopamine receptor genes, *dop-1*, *dop-2*, and *dop-3*, are downregulated in L4-stage animals, confirming that the decreased levels previously reported in L1-stage animals (Felton and Johnson 2011) remain low in animals at the stage used for our behavior assays.

Like the mammalian VMATs, CAT-1 mediates the packaging and transport of the biogenic amines into synaptic vesicles and is required for proper release of dopamine from presynaptic neurons in *C. elegans* (Duerr *et al.* 1999). The dopamine transporter, DAT-1, is localized to the synapses of all dopaminergic neurons of *C. elegans* males and hermaphrodites (McDonald *et al.* 2007) and is responsible for neurotransmitter clearance from the synaptic cleft (Carvelli *et al.* 2004; Torres *et al.* 2003). In otherwise wild-type animals, loss of *dat-1* leads to increased activation of the DOP-3 receptors located on cholinergic motor neurons (Chase *et al.* 2004). Consequently, *dat-1* animals are paralyzed in water as a result of DOP-3 hyperactivation; this behavior can be measured using a SWIP assay (Chase and Koelle 2007; McDonald *et al.* 2007). SWIP does not occur in *cat-1* animals because dopamine is not efficiently packaged or subsequently released into the synaptic cleft. We reasoned that if *hlh-17(ns204)* animals synthesize and release normal levels of dopamine, but produce less DAT-1, then they would be less efficient than wild-type animals at clearing extrasynpatic dopamine from the synaptic cleft. To test this hypothesis, we conducted SWIP assays with wild-type, *hlh-17(ns204)*, and *dat-1(ok157)* animals. As reported previously (McDonald *et al.* 2007), and as shown in Figure 2, *dat-1(ok157)* animals, but not wild-type animals, have a strong SWIP response after 20 min in water. The SWIP response of *hlh-17(ns204)* animals was an intermediate response, with approximately 40% of the animals becoming paralyzed after 20 min in water. This phenotype was rescued by transgenic expression of HLH-17. The result suggests that loss of HLH-17 compromises the ability of mutant animals to clear dopamine from the synaptic cleft and could be interpreted as representing a slight, rather than complete, loss of *dat-1* activity.

The SWIP phenotype seen in *dat-1* animals is completely rescued by loss of DOP-3 (Sugiura *et al.* 2005); hence, we reasoned that the reduced SWIP response of *hlh-17(ns204)* animals, which is an intermediate of the responses of wild-type and *dat-1* animals, may be the result of having decreased levels of both *dop-3* and *dat-1*. To test this hypothesis, we compared the SWIP phenotypes of *hlh-17(ns204)*; *dat-1(ok157)* animals and of *hlh-17(ns204)*; *dop-3(vs106)* animals with those of *dat-1(ok157)* and *dop-3(vs106)* animals, respectively. As shown in Figure 2, complete loss of *dat-1* activity enhanced the SWIP response of *hlh-17(ns204)* animals, whereas complete loss of *dop-3* activity significantly decreased the SWIP response. Furthermore, the SWIP phenotypes of *hlh-17(ns204)*; *dop-3(vs106)* animals and *hlh-17(ns204)*; *dop-3(vs106)*; *dat-1(ok157)* animals were not significantly different from that of *dop-3(vs106)* animals (*P* = 0.574 and 0.265, respectively). Interestingly, loss of *hlh-17* and *dat-1* appears to be an additive effect: a comparison of the differences of the means shows that the difference for wild-type *vs. hlh-17*; *dat-1* is equal to the sum of the differences for wild-type *vs. hlh-17* and wild-type *vs. dat-1*. These results underscore the dependence of the SWIP phenotype on DOP-3. Furthermore, the results suggest that the SWIP response is not mediated solely through *dat-1*, and that *hlh-17* may affect the SWIP phenotype through both *dat-1*-dependent and *dat-1*-independent mechanisms. A *dat-1*-independent, *dop-3*-dependent mechanism for the SWIP phenotype is consistent with the results of a previously reported forward genetics screen (Hardaway *et al.* 2012) and suggests that the HLH-17 transcriptional network may include genes that act in parallel to *dat-1*.

Our results from the dopamine paralysis assays and the egg-laying assays suggest that *hlh-17(ns204)* animals are less sensitive to exogenous dopamine, a result that is consistent with reduced *dop-3* activity. The results from assays for BSR and SWIP, both of which rely on normal synthesis and release of endogenous dopamine from presynaptic neurons, suggest that *hlh-17(ns204)* animals produce normal amounts of dopamine but are deficient in the ability to transport the dopamine. This result is also consistent with reduced *dop-3* activity. Likewise, a failure in the ability to transport dopamine from the synaptic cleft is consistent with reduced *dat-1* activity, as is the SWIP phenotype of *hlh-17(ns204)* animals. From these results, we conclude that HLH-17 functions to control extrasynpatic dopamine levels, in part by its regulation of *dop-3* and *dat-1*.

### The *hlh-17* mutants are responsive to reuptake inhibitors that are selective for dopamine, but not for serotonin

**Figure 3:**
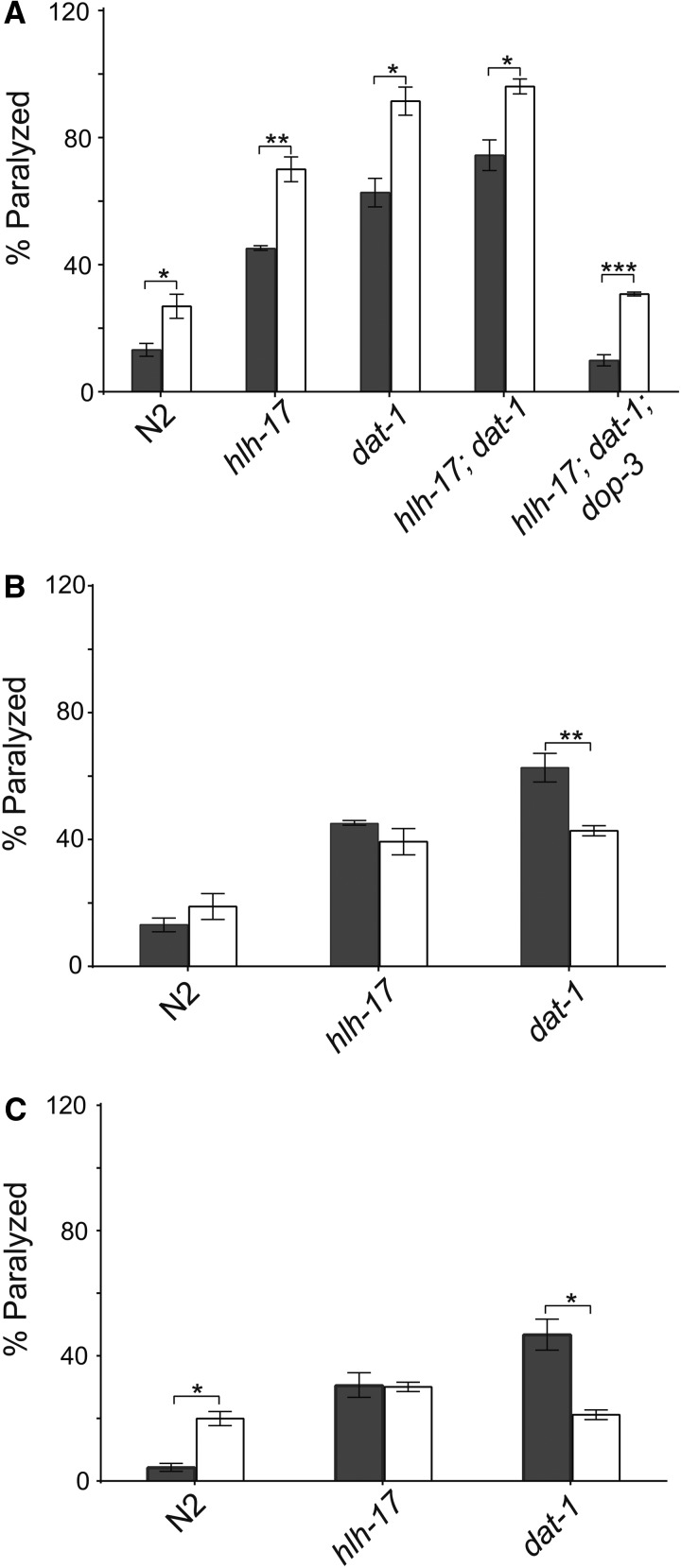
The *hlh-17* animals respond selectively to reuptake inhibitors. (A) Pretreatment with the DAT reuptake inhibitor, bupropion, increases the SWIP phenotype of N*2*, *hlh-17(ns204)*, *dat-1(ok157)*, and *hlh-17(ns204)*; *dop-3(vs106)* animals. The ability of bupropion to enhance SWIP behavior is not dependent on DOP-3. The SWIP phenotype in hlh-17(ns204) animals is unaffected by pretreatment with reserpine (B) or fluoxetine (C). In all panels, n = 30 animals/rep/strain; dark bars = minus inhibitor; and light bars = plus inhibitor. *P < 0.05; **P < 0.005; ***P < 0.0005.

Bupropion is a selective norepinephrine and dopamine reuptake inhibitor commonly used in mice and human studies (Dellagioia *et al.* 2012; Roelands *et al.* 2012; Rosenberg *et al.* 2013) and in the treatment of ADHD (Cantwell 1998; Reimherr *et al.* 2005) and depression (Carlat 2012; Stahl *et al.* 2013). Reuptake inhibitors block the ability of a transporter to move a neurotransmitter from the synapse to the presynaptic neuron or the surrounding glial cells, thereby increasing extracellular concentrations that ultimately increase neurotransmission. We reasoned that the intermediate SWIP behavior of *hlh-17(ns204)* animals occurs because these animals still produce a small amount of functional DAT-1, and that treatment with bupropion would increase SWIP in *hlh-17(ns204)* animals. As expected, pretreatment of *hlh-17(ns204)* animals with bupropion increased their SWIP response to that of untreated *dat-1(ok157)* animals (Figure 3), supporting our mRNA studies showing that *dat-1* expression is reduced but not completely eliminated in *hlh-17(ns204)* animals. It has been shown previously that SWIP can be rescued in *dat-1(ok157)* animals by pretreatment with the dopamine antagonist reserpine (McDonald *et al.* 2007), an antipsychotic drug that depletes vesicular dopamine stores by blocking the vesicular monoamine transporter (VMAT). As shown in Figure 3, pretreatment with reserpine reduced the SWIP responses of *dat-1(ok157)* animals but did not affect SWIP in wild-type animals or in *hlh-17(ns204)* animals. This result was unexpected because *cat-1* mRNA levels are not affected in *hlh-17(ns204)* animals; however, others have reported reserpine insensitive mutants that show SWIP behavior in a *dat-1*-dependent manner (Hardaway *et al.* 2012). Bupropion pretreatment also increased SWIP in *dat-1(ok157)* animals, *hlh-17(ns204)*; *dat-1(ok157)* animals, and *hlh-17(ns204)*; *dat-1(ok157)*; *dop-3(vs106)* animals (Figure 3). Together, these results further emphasize that SWIP behavior may not be mediated solely through dopamine reuptake by DAT-1. The ability to induce SWIP behavior in *dop-3* animals suggests that the mechanism may occur through a dopamine-independent mechanism.

To test the possibility that the SWIP phenotype is also modulated through serotonin, although a role for 5HT during SWIP has not been reported to date, we measured the SWIP response of WT, *hlh-17(ns204)*, and *dat-1(ok157)* animals after exposure to fluoxetine. Fluoxetine blocks the function of SERT/MOD-5, the serotonin (5HT) reuptake transporter (Keowkase *et al.* 2010; Kullyev *et al.* 2010). As seen in Figure 3, the SWIP response phenotype increased in wild-type animals that were pretreated with fluoxetine but decreased in similarly treated *dat-1*(ok157) animals. These results can be explained by the action of fluoxetine, which is known to increase extracellular concentrations of dopamine (Bymaster *et al.* 2002; Koch *et al.* 2002). The excess dopamine in treated wild-type animals would phenocopy mutants that have increased extracellular levels of dopamine and have an increased SWIP response. Fluoxetine can also aggressively inhibit any transport of dopamine by the serotonin transporters (Bymaster *et al.* 2002) so that treated *dat-1* animals would show a reduced SWIP response, analogous to the reduced response of *dat-1*; *dop-3* animals (McDonald *et al.*, 2007). Interestingly, *hlh-17(ns204)* animals were insensitive to fluoxetine, although they have normal levels of *mod-5* mRNA (see Figure 2) and respond to exogenous serotonin in egg-laying assays (Felton and Johnson 2011). Fluoxetine has previously been shown to act via both serotonin-dependent and serotonin-independent mechanisms in *C. elegans* (Kullyev *et al.* 2010; Ranganathan *et al.* 2001). In future studies we will further explore the role of HLH-17 in serotonin signaling, which may also address the mechanisms of fluoxetine resistance in *hlh-17(ns204)* animals.

### The *hlh-17(ns204)* animals are not defective in acetylcholine release

It is possible that the SWIP response of *hlh-17(ns204)* animals is insensitive to both reserpine and fluoxetine because HLH-17 influences the activity of *C. elegans* biogenic amines in a manner that, with the exception of dopamine, does not involve the regulation of genes directly involved in neurotransmitter synthesis, packaging, or transport. A more attractive, alternative possibility is that HLH-17 influences acetylcholine release, as the phenotypic effects of both reserpine (Saharia *et al.* 2012) and fluoxetine (Bolanos *et al.* 2002; Chau *et al.* 2011) are dependent on acetylcholine. In support of this possibility, the inhibitory effect of fluoxetine on acetylcholine release in rats is dependent on activity of the dopaminergic D2 receptors (Bolanos *et al.* 2002). Furthermore, loss of *dop-3* activity in *C. elegans* has recently been shown to increase acetylcholine release, whereas null alleles of genes required for acetylcholine release have been shown to rescue the SWIP phenotype in *dat-1(ok157)* animals (Allen *et al.* 2011).

**Figure 4:**
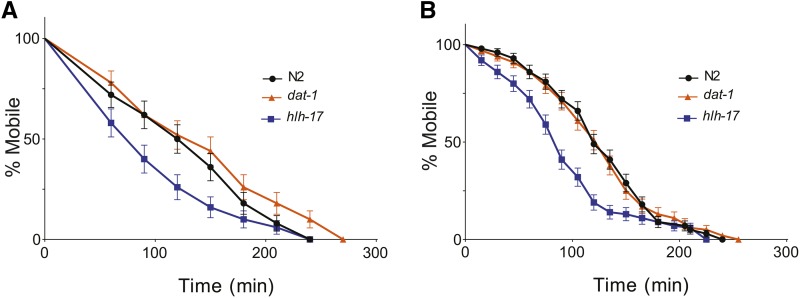
The hlh-17 animals do not have reduced acetylcholine signaling. (A) *hlh-17(ns204)* animals are more susceptible to aldicarb-induced paralysis than wild-type (*P* = 0.0428) and *dat-1(ok157)* animals (*P* = 0.1319). (B) The *hlh-17(ns204)* animals are more susceptible to levamisole-induced paralysis than wild-type (*P* = 0.0002) and *dat-1(ok157)* animals (*P* = 0.0002). In all panels, n = 30 animals/rep/strain.

We used aldicarb and levamisole sensitivity assays to examine acetylcholine release and acetylcholine reception, respectively, in *hlh-17(ns204)* animals. Aldicarb is an acetylcholinesterase inhibitor and thereby increases the concentration of acetylcholine in the neuromuscular junction. Animals with reduced acetylcholine release are resistant to aldicarb-induced paralysis, whereas those with increased acetylcholine release are more sensitive (Allen *et al.* 2011; Rand 2007). As shown in Figure 4, *hlh-17(ns204)* animals are more sensitive to aldicarb than wild-type and *dat-1(ok157)* animals (*P* = 0.0428 and 0.132, respectively). This result is consistent with the weak effects of the *dop-3(v106)* mutation on aldicarb sensitivity that was previously reported, and suggests that acetylcholine release is otherwise normal in *hlh-17(ns204)* animals. We also found that *hlh-17(ns204)* animals are more sensitive to levamisole (*P* = 0.0002), a cholinergic agonist that binds selectively to acetylcholine receptors in body-wall muscles (Rand 2007). We are able to tentatively explain this increased sensitivity based on our unpublished microarray analysis that indicates that the activity of the nicotinic acetylcholine receptor gene, *lev-8*, is upregulated in *hlh-17(ns204)* animals. Interestingly, our microarray data indicated that the gene encoding the calcium/calmodulin-dependent protein kinase UNC-43 is also upregulated. Mutants carrying gain-of-function alleles of *unc-43* have previously been reported to have increased resistance to fluoxetine. As shown in Figure 2, we were able to validate these results using RT-qPCR analysis. mRNA levels of *unc-43* and *lev-8* are increased in *hlh-17* animals, whereas the level of *mod-5*, a gene that was not differentially affected in our microarray analysis, remained unaffected. To our knowledge, loss of dopamine receptor activity, in particular *dop-3*, has not been tested; however, animals that are defective in dopamine synthesis display normal sensitivity to levamisole (Suo and Ishiura 2013). Taken together, our results suggest that neither acetylcholine release nor acetylcholine reception is compromised in *hlh-17(ns204)* animals, and that the resistance to reserpine and fluoxetine may be mediated through other genes in the HLH-17 transcriptional network.

## Conclusion

The Olig sub-family of bHLH transcription factors influences the specification of oligodendrocytes, myelin formation, and axon pathfinding of motor neurons in both invertebrates and vertebrates (Lu *et al.* 2002; Oyallon *et al.* 2012; Tiso *et al.* 2009; Zhou and Anderson 2002). In *C. elegans*, HLH-17 is an Olig homolog that is expressed in sheath cells of the dopaminergic neurons; however, this protein has no known role in glial cell specification, neurite extension, or axon guidance (Yoshimura *et al.* 2008). The work presented here and in previous studies points to a role for HLH-17 in controlling dopamine-dependent behaviors. Specifically, our work suggests that HLH-17 is needed to clear extracellular dopamine from the synaptic cleft. First, *hlh-17(ns204)* animals have reduced mRNA levels for *dat-1*, *dop-3*, *dop-2*, and *dop-1* but maintain normal levels of *cat-1* and *cat-2*. Second, the SWIP response of *hlh-17(ns204)* animals is consistent with reduced levels of *dat-1* and *dop-3* and is rescued when *dop-3* activity is completely eliminated. Third, *hlh-17(ns204)* animals are not defective in acetylcholine release and, in fact, show an increased sensitivity to aldicarb that is consistent with the increased acetylcholine release that occurs in animals with reduced *dop-3* activity.

The bHLH transcription factor family has well-established roles in neurogenesis and the specification and maintenance of neuronal identity. In *Drosophila*, for example, the bHLH gene, lethal of scute, is required for cell-specific transcription of the dopaminergic H-cell neuron of the ventral nerve cord and for specification of the non-midline dopaminergic neurons (Oyallon *et al.* 2012; Stagg *et al.* 2011) In zebrafish, Olig2 regulates expression of the gene encoding Sim1, a bHLH-PAS protein that drives specification of the diencephalic dopaminergic neurons (Borodovsky *et al.* 2009). Less clear, however, is whether HLH-17 plays a conserved role in the regulation of genes required for neurotransmitter signaling in general and dopamine signaling in particular. The gene encoding the human dopamine reuptake transporter is regulated by the hairy/enhancer of split-like bHLH protein, HesR1. HesR1 represses activity of the human DAT1 gene in cell culture by binding to sequences in the 3′ UTR(Fuke *et al.* 2005). HesR1 also affects dopamine receptor expression in mice, and hesr1 mutant mice show defects in dopamine-dependent behaviors (Fuke *et al.* 2006). Although both are basic helix-loop-helix proteins, HLH-17 shows no sequence similarity to HesR1 and is most similar to the human olig-related proteins, bHLHb5/Beta3 and bHLHb4. Neither of these proteins has been shown to directly regulate expression of the dopamine transporter or dopamine receptor genes in humans. However, both proteins are part of the bHLH transcriptional network that drives retina development (Feng *et al.* 2006; Pennesi *et al.* 2006; Skowronska-Krawczyk *et al.* 2004), and the dopamine receptors are critical for normal retinal function (He *et al.* 2013; Nguyen-Legros *et al.* 1999; Ogata *et al.* 2012; Reis *et al.* 2007; Yang *et al.* 2013). Our own transgenic expression data show strong expression of *hlh-17* in the cephalic sheath cells of wild-type animals and, on its own, do not support the direct regulation of *dat-1* and *dop-3* by HLH-17. However, mRNA for both *dop-3* and *hlh-17* was recently detected in glutamate receptor neurons of L2-stage animals and in the cephalic sheath cells of young adult animals (Spencer *et al.* 2011; see also WormViz). Furthermore, mRNA for *dop-3*, *dat-1*, and *hlh-17* was detected in the dopamine neurons and panneuronal neurons of late embryos and L2-stage animals, respectively. Taken together with the epistasis analysis presented in this study, the colocalization of these mRNAs supports the possibility that HLH-17 is a direct regulator of *dop-3* and *dat-1*. However, further studies are in progress to confirm that prediction.

Interestingly, the SWIP response in *hlh-17(ns204)* animals is enhanced by pre-treatment with bupropion, an antidepressant and DAT inhibitor that is used to treat ADHD in adults and children (Faraone and Glatt 2010; Jafarinia *et al.* 2012) but is unaffected by the antidepressant fluoxetine and the dopamine antagonist, reserpine. This finding underscores the need to develop animal models of dopamine signaling that accurately reflect the effects of reduced expression of multiple neurotransmitter signaling pathway genes, rather than complete loss of function of a single gene. Our future studies are aimed at exploiting *hlh-17(ns204)* for this purpose.

## References

[bib1] Allen A. T. Maher K. N. Wani K. A. Betts K. E. Chase D. L., 2011 Coexpressed D1- and D2-like dopamine receptors antagonistically modulate acetylcholine release in Caenorhabditis elegans. Genetics 188: 579–590 21515580 10.1534/genetics.111.128512 PMC3176529

[bib2] Biedermann B. Frohlich E. Grosche J. Wagner H. J. Reichenbach A., 1995 Mammalian Muller (glial) cells express functional D2 dopamine receptors. Neuroreport 6: 609–612 7605910 10.1097/00001756-199503000-00006

[bib3] Bolanos C. A. Trksak G. H. Cohen O. S. Jackson D., 2002 Differential serotonergic inhibition of in vitro striatal [^3^H]acetylcholine release in prenatally cocaine-exposed male and female rats. Prog. Neuropsychopharmacol. Biol. Psychiatry 26: 1339–1348 12502023 10.1016/s0278-5846(02)00299-3

[bib4] Borodovsky N. Ponomaryov T. Frenkel S. Levkowitz G., 2009 Neural protein Olig2 acts upstream of the transcriptional regulator Sim1 to specify diencephalic dopaminergic neurons. Dev. Dyn. 238: 826–834. 10.1002/dvdy.21894 19253397

[bib5] Brenner S., 1974 The genetics of Caenorhabditis elegans. Genetics 77: 71–94 4366476 10.1093/genetics/77.1.71 PMC1213120

[bib6] Bymaster F. P. Zhang W. Carter P. A. Shaw J. Chernet E., 2002 Fluoxetine, but not other selective serotonin uptake inhibitors, increases norepinephrine and dopamine extracellular levels in prefrontal cortex. Psychopharmacology (Berl.) 160: 353–361 11919662 10.1007/s00213-001-0986-x

[bib7] Cantwell D. P., 1998 ADHD through the life span: the role of bupropion in treatment. J. Clin. Psychiatry 59(Suppl 4): 92–94 9554326

[bib8] Carlat D., 2012 Evidence-based somatic treatment of depression in adults. Psychiatr. Clin. North Am. 35: 131–142 22370495 10.1016/j.psc.2011.11.002

[bib9] Carvelli L. McDonald P. W. Blakely R. D. Defelice L. J., 2004 Dopamine transporters depolarize neurons by a channel mechanism. Proc. Natl. Acad. Sci. USA 101: 16046–16051 15520385 10.1073/pnas.0403299101 PMC528740

[bib10] Chase D. L. Koelle M. R., 2007 Biogenic amine neurotransmitters in C. elegans. WormBook Feb 20: 1–15 18050501 10.1895/wormbook.1.132.1 PMC4781333

[bib11] Chase D. L. Pepper J. S. Koelle M. R., 2004 Mechanism of extrasynaptic dopamine signaling in Caenorhabditis elegans. Nat. Neurosci. 7: 1096–1103 15378064 10.1038/nn1316

[bib12] Chau D. T. Rada P. V. Kim K. Kosloff R. A. Hoebel B. G., 2011 Fluoxetine alleviates behavioral depression while decreasing acetylcholine release in the nucleus accumbens shell. Neuropsychopharmacology 36: 1729–1737. 10.1038/npp.2011.54 PMC3138650 21525864

[bib13] Chen L. Krause M. Sepanski M. Fire A., 1994 The Caenorhabditis elegans MYOD homologue HLH-1 is essential for proper muscle function and complete morphogenesis. Development 120: 1631–1641 8050369 10.1242/dev.120.6.1631

[bib14] Choi Y. K. Tarazi F. I., 2010 Alterations in dopamine and glutamate neurotransmission in tetrahydrobiopterin deficient spr−/− mice: relevance to schizophrenia. BMB Rep 43: 593–598 20846490 10.5483/BMBRep.2010.43.9.593

[bib15] Dellagioia N. Devine L. Pittman B. Hannestad J., 2012 Bupropion pre-treatment of endotoxin-induced depressive symptoms. Brain Behav. Immun. 31: 197–204 10.1016/j.bbi.2012.10.008 23064079

[bib16] Dempsey C. M. Mackenzie S. M. Gargus A. Blanco G. Sze J. Y., 2005 Serotonin (5HT), fluoxetine, imipramine and dopamine target distinct 5HT receptor signaling to modulate Caenorhabditis elegans egg-laying behavior. Genetics 169: 1425–1436 15654117 10.1534/genetics.104.032540 PMC1449529

[bib17] Doonan R. Hatzold J. Raut S. Conradt B. Alfonso A., 2008 HLH-3 is a C. elegans Achaete/Scute protein required for differentiation of the hermaphrodite-specific motor neurons. Mech. Dev. 125: 883–893 18586090 10.1016/j.mod.2008.06.002

[bib18] Duerr J. S. Frisby D. L. Gaskin J. Duke A. Asermely K., 1999 The cat-1 gene of Caenorhabditis elegans encodes a vesicular monoamine transporter required for specific monoamine-dependent behaviors. J. Neurosci. 19: 72–84. 10.1523/JNEUROSCI.19-01-00072.1999 PMC6782383 9870940

[bib19] Faraone S. V. Glatt S. J., 2010 A comparison of the efficacy of medications for adult attention-deficit/hyperactivity disorder using meta-analysis of effect sizes. J. Clin. Psychiatry 71: 754–763 20051220 10.4088/JCP.08m04902pur

[bib20] Felton C. M. Johnson C. M., 2011 Modulation of dopamine-dependent behaviors by the Caenorhabditis elegans Olig homolog HLH-17. J. Neurosci. Res. 89: 1627–1636 21688290 10.1002/jnr.22694

[bib21] Feng L. Xie X. Joshi P. S. Yang Z. Shibasaki K., 2006 Requirement for Bhlhb5 in the specification of amacrine and cone bipolar subtypes in mouse retina. Development 133: 4815–4825 17092954 10.1242/dev.02664 PMC2992969

[bib22] Frank C. A. Baum P. D. Garriga G., 2003 HLH-14 is a C. elegans achaete-scute protein that promotes neurogenesis through asymmetric cell division. Development 130: 6507–6518 14627726 10.1242/dev.00894

[bib23] Fuke S. Minami N. Kokubo H. Yoshikawa A. Yasumatsu H., 2006 Hesr1 knockout mice exhibit behavioral alterations through the dopaminergic nervous system. J. Neurosci. Res. 84: 1555–1563 16998899 10.1002/jnr.21062

[bib24] Fuke S. Sasagawa N. Ishiura S., 2005 Identification and characterization of the Hesr1/Hey1 as a candidate trans-acting factor on gene expression through the 3′ non-coding polymorphic region of the human dopamine transporter (DAT1) gene. J. Biochem. 137: 205–216 15749835 10.1093/jb/mvi020

[bib25] Hallam S. Singer E. Waring D. Jin Y., 2000 The C. elegans NeuroD homolog cnd-1 functions in multiple aspects of motor neuron fate specification. Development 127: 4239–4252 10976055 10.1242/dev.127.19.4239

[bib26] Hardaway J. A. Hardie S. L. Whitaker S. M. Baas S. R. Zhang B., 2012 Forward genetic analysis to identify determinants of dopamine signaling in Caenorhabditis elegans using swimming-induced paralysis. G3 (Bethesda) **2:** 961–975. 10.1534/g3.112.003533 PMC3411251 22908044

[bib27] Harfe B. D. Vaz Gomes A. Kenyon C. Liu J. Krause M., 1998 Analysis of a Caenorhabditis elegans Twist homolog identifies conserved and divergent aspects of mesodermal patterning. Genes Dev. 12: 2623–2635 9716413 10.1101/gad.12.16.2623 PMC317087

[bib28] He Q. Xu H. P. Wang P. Tian N., 2013 Dopamine D1 receptors regulate the light dependent development of retinal synaptic responses. PLoS ONE 8: e79625 24260267 10.1371/journal.pone.0079625 PMC3834122

[bib29] Jafarinia M. Mohammadi M. R. Modabbernia A. Ashrafi M. Khajavi D., 2012 Bupropion *vs.* methylphenidate in the treatment of children with attention-deficit/hyperactivity disorder: randomized double-blind study. Hum. Psychopharmacol. 27: 411–418 22806822 10.1002/hup.2242

[bib30] Keowkase R. Aboukhatwa M. Luo Y., 2010 Fluoxetine protects against amyloid-beta toxicity, in part via daf-16 mediated cell signaling pathway, in Caenorhabditis elegans. Neuropharmacology 59: 358–365 20420844 10.1016/j.neuropharm.2010.04.008 PMC2926186

[bib31] Koch S. Perry K. W. Nelson D. L. Conway R. G. Threlkeld P. G., 2002 R-fluoxetine increases extracellular DA, NE, as well as 5-HT in rat prefrontal cortex and hypothalamus: an in vivo microdialysis and receptor binding study. Neuropsychopharmacology **27:** 949–959. 10.1016/S0893-133X(02)00377-9 12464452

[bib32] Krause M. Park M. Zhang J. M. Yuan J. Harfe B., 1997 A C. elegans E/Daughterless bHLH protein marks neuronal but not striated muscle development. Development 124: 2179–2189 9187144 10.1242/dev.124.11.2179

[bib33] Kullyev A. Dempsey C. M. Miller S. Kuan C. J. Hapiak V. M., 2010 A genetic survey of fluoxetine action on synaptic transmission in Caenorhabditis elegans. Genetics 186: 929–941 20739712 10.1534/genetics.110.118877 PMC2975281

[bib34] Kuric E. Wieloch T. Ruscher K., 2013 Dopamine receptor activation increases glial cell line-derived neurotrophic factor in experimental stroke. Exp. Neurol. 247: 202–208 23664961 10.1016/j.expneurol.2013.04.016

[bib67] Lewis J. A. Wu C. H. Berg H. Levine J. H., 1980 The genetics of levamisole resistance in the nematode *Caenorhabditis elegans*. Genetics 95: 905–928 7203008 10.1093/genetics/95.4.905 PMC1214276

[bib35] Ligon K. L. Fancy S. P. Franklin R. J. Rowitch D. H., 2006 Olig gene function in CNS development and disease. Glia 54: 1–10 16652341 10.1002/glia.20273

[bib36] Lu Q. R. Sun T. Zhu Z. Ma N. Garcia M., 2002 Common developmental requirement for Olig function indicates a motor neuron/oligodendrocyte connection. Cell 109: 75–86 11955448 10.1016/s0092-8674(02)00678-5

[bib69] Mahoney T. R. Luo S. Monet M. L., 2006 Analysis of synaptic transmission in *Caenorhabditis elegans* using an alicarb-sensitivity assay. Nat Protoc. 1: 1772–1777 17487159 10.1038/nprot.2006.281

[bib37] McDonald P. W. Hardie S. L. Jessen T. N. Carvelli L. Matthies D. S., 2007 Vigorous motor activity in Caenorhabditis elegans requires efficient clearance of dopamine mediated by synaptic localization of the dopamine transporter DAT-1. J. Neurosci. 27: 14216–14227 18094261 10.1523/JNEUROSCI.2992-07.2007 PMC6673513

[bib38] McMiller T. L. Johnson C. M., 2005 Molecular characterization of HLH-17, a C. elegans bHLH protein required for normal larval development. Gene 356: 1–10 16014321 10.1016/j.gene.2005.05.003 PMC2040385

[bib39] Middleton L. S. Apparsundaram S. King-Pospisil K. A. Dwoskin L. P., 2007 Nicotine increases dopamine transporter function in rat striatum through a trafficking-independent mechanism. Eur. J. Pharmacol. 554: 128–136 17141211 10.1016/j.ejphar.2006.09.074 PMC1920186

[bib40] Missale C. Nash S. R. Robinson S. W. Jaber M. Caron M. G., 1998 Dopamine receptors: from structure to function. Physiol. Rev. 78: 189–225 9457173 10.1152/physrev.1998.78.1.189

[bib68] Nguyen M. Alfonso A. Johnson C. D. Rand J. B., 1995 *Caenorhabditis elegans* mutants resistant to inhibitors of acetylcholinesterase. Genetics 140: 527–535 7498734 10.1093/genetics/140.2.527 PMC1206632

[bib41] Nguyen-Legros J. Versaux-Botteri C. Vernier P., 1999 Dopamine receptor localization in the mammalian retina. Mol. Neurobiol. 19: 181–204 10495103 10.1007/BF02821713

[bib42] Ogata G. Stradleigh T. W. Partida G. J. Ishida A. T., 2012 Dopamine and full-field illumination activate D1 and D2–D5-type receptors in adult rat retinal ganglion cells. J. Comp. Neurol. 520: 4032–4049 22678972 10.1002/cne.23159 PMC3538137

[bib43] Oyallon J. Apitz H. Miguel-Aliaga I. Timofeev K. Ferreira L., 2012 Regulation of locomotion and motoneuron trajectory selection and targeting by the Drosophila homolog of Olig family transcription factors. Dev. Biol. 369: 261–276 22796650 10.1016/j.ydbio.2012.06.027 PMC3464432

[bib44] Pennesi M. E. Bramblett D. E. Cho J. H. Tsai M. J. Wu S. M., 2006 A role for bHLH transcription factors in retinal degeneration and dysfunction. Adv. Exp. Med. Biol. 572: 155–161 17249569 10.1007/0-387-32442-9_23

[bib45] Poole R. J. Bashllari E. Cochella L. Flowers E. B. Hobert O., 2011 A genome-wide RNAi screen for factors involved in neuronal specification in Caenorhabditis elegans. PLoS Genet. 7: e1002109 21698137 10.1371/journal.pgen.1002109 PMC3116913

[bib46] Rand, J. B., 2007 Acetylcholine in *C. elegans* WormBook Jan 30: 1–21 10.1895/wormbook.1.131.1 PMC4781110 18050502

[bib47] Ranganathan R. Sawin E. R. Trent C. Horvitz H. R., 2001 Mutations in the Caenorhabditis elegans serotonin reuptake transporter MOD-5 reveal serotonin-dependent and -independent activities of fluoxetine. J. Neurosci. 21: 5871–5884. 10.1523/JNEUROSCI.21-16-05871.2001 PMC6763176 11487610

[bib48] Reimherr F. W. Hedges D. W. Strong R. E. Marchant B. K. Williams E. D., 2005 Bupropion SR in adults with ADHD: a short-term, placebo-controlled trial. Neuropsychiatr. Dis. Treat. 1: 245–251 18568102 PMC2416755

[bib49] Reis R. A. Ventura A. L. Kubrusly R. C. de Mello M. C. de Mello F. G., 2007 Dopaminergic signaling in the developing retina. Brain Res. Brain Res. Rev. 54: 181–188 10.1016/j.brainresrev.2007.01.001 17292477

[bib50] Rieckher M. Kourtis N. Pasparaki A. Tavernarakis N., 2009 Transgenesis in Caenorhabditis elegans. Methods Mol. Biol. 561: 21–39 19504062 10.1007/978-1-60327-019-9_2

[bib51] Roelands B. Watson P. Cordery P. Decoster S. Debaste E., 2012 A dopamine/noradrenaline reuptake inhibitor improves performance in the heat, but only at the maximum therapeutic dose. Scand. J. Med. Sci. Sports 22: e93–e98 22845895 10.1111/j.1600-0838.2012.01502.x

[bib52] Rosenberg M. B. Carroll F. I. Negus S. S., 2013 Effects of monoamine reuptake inhibitors in assays of acute pain-stimulated and pain-depressed behavior in rats. J. Pain 14: 246–259 23332494 10.1016/j.jpain.2012.11.006 PMC3743421

[bib53] Saharia K. Arya U. Kumar R. Sahu R. Das C. K., 2012 Reserpine modulates neurotransmitter release to extend lifespan and alleviate age-dependent Abeta proteotoxicity in Caenorhabditis elegans. Exp. Gerontol. 47: 188–197 22212533 10.1016/j.exger.2011.12.006

[bib54] Skowronska-Krawczyk D. Ballivet M. Dynlacht B. D. Matter J. M., 2004 Highly specific interactions between bHLH transcription factors and chromatin during retina development. Development 131: 4447–4454 15342472 10.1242/dev.01302

[bib55] Spencer W. C. Zeller G. Watson J. D. Henz S. R. Watkins K. L., 2011 A spatial and temporal map of C. elegans gene expression. Genome Res. 21: 325–341 21177967 10.1101/gr.114595.110 PMC3032935

[bib56] Stagg S. B. Guardiola A. R. Crews S. T., 2011 Dual role for Drosophila lethal of scute in CNS midline precursor formation and dopaminergic neuron and motoneuron cell fate. Development 138: 2171–2183 21558367 10.1242/dev.056507 PMC3091489

[bib57] Stahl S. M. Lee-Zimmerman C. Cartwright S. Morrissette D. A., 2013 Serotonergic drugs for depression and beyond. Curr. Drug Targets 14: 578–585 23531115 10.2174/1389450111314050007

[bib58] Sugiura M. Fuke S. Suo S. Sasagawa N. Van Tol H. H., 2005 Characterization of a novel D2-like dopamine receptor with a truncated splice variant and a D1-like dopamine receptor unique to invertebrates from Caenorhabditis elegans. J. Neurochem. 94: 1146–1157 16001968 10.1111/j.1471-4159.2005.03268.x

[bib59] Suo S. Ishiura S., 2013 Dopamine modulates acetylcholine release via octopamine and CREB signaling in Caenorhabditis elegans. PLoS ONE 8: e72578 23977320 10.1371/journal.pone.0072578 PMC3745381

[bib60] Tamai K. K. Nishiwaki K., 2007 bHLH transcription factors regulate organ morphogenesis via activation of an ADAMTS protease in C. elegans. Dev. Biol. 308: 562–571 17588558 10.1016/j.ydbio.2007.05.024

[bib61] Tiso N. Filippi A. Benato F. Negrisolo E. Modena N., 2009 Differential expression and regulation of olig genes in zebrafish. J. Comp. Neurol. 515: 378–396 19425111 10.1002/cne.22054

[bib62] Torres G. E. Carneiro A. Seamans K. Fiorentini C. Sweeney A., 2003 Oligomerization and trafficking of the human dopamine transporter. Mutational analysis identifies critical domains important for the functional expression of the transporter. J. Biol. Chem. 278: 2731–2739 12429746 10.1074/jbc.M201926200

[bib63] Xie W. Li X. Li C. Zhu W. Jankovic J., 2010 Proteasome inhibition modeling nigral neuron degeneration in Parkinson’s disease. J. Neurochem. 115: 188–199 20649845 10.1111/j.1471-4159.2010.06914.x

[bib64] Yang J. Pahng J. Wang G. Y., 2013 Dopamine modulates the off pathway in light-adapted mouse retina. J. Neurosci. Res. 91: 138–150 23023788 10.1002/jnr.23137

[bib65] Yoshimura S. Murray J. I. Lu Y. Waterston R. H. Shaham S., 2008 mls-2 and vab-3 Control glia development, hlh-17/Olig expression and glia-dependent neurite extension in C. elegans. Development 135: 2263–2275 18508862 10.1242/dev.019547

[bib66] Zhou Q. Anderson D. J., 2002 The bHLH transcription factors OLIG2 and OLIG1 couple neuronal and glial subtype specification. Cell 109: 61–73 11955447 10.1016/s0092-8674(02)00677-3

